# IL-15 is an inflammatory mediator in the tumor microenvironment

**DOI:** 10.1186/2051-1426-3-S2-P417

**Published:** 2015-11-04

**Authors:** Scott M Anthony, Gabriel Pham, Kimberly S Schluns

**Affiliations:** 1 University of Texas MD Anderson Cancer Center/Department of Immunology/The University of Texas Graduate School of Biomedical Sciences at Houston, Houston, TX, USA; 2 University of Cincinnati College of Medicine, Cincinnati, OH, USA; 3 The University of Texas MD Anderson Cancer Center/Department of Immunology, Houston, TX, USA

## 

Previous studies have shown that activation of the Stimulator of Interferon Genes (STING) pathway is important for regulating type I Interferons (IFN) in tumors. Furthermore, tumor DNA induces IL-15 and IL-15Rα mRNA in cultured dendritic cells (DC) in a STING-dependent manner. Since we have recently shown type I IFNs induce soluble IL-15Rα/IL-15 complexes (sIL-15 complexes), we set out to determine if sIL-15 complexes are produced in tumor microenvironment and are regulated by STING signaling. To determine if STING induces sIL-15 complexes, mice were given STING agonists (c-di-GMP) either i.v. or i.p. One day later, sIL-15 complexes were increased in serum and splenic homogenates of STING-treated mice. In addition, STING agonists directly induced sIL-15 complexes in BM-derived DCs. To examine sIL-15 complexes in the tumor microenvironment, B16-F10 tumors of various sizes were isolated from mice and sIL-15 complexes were measured in homogenates from tumors, draining lymph nodes, and spleen. Interestingly, levels of sIL-15 complexes were high in homogenates from small tumors (less than 100mm^2^) and their draining lymph nodes but low in larger tumors. These findings suggest IL-15 is an inflammatory factor produced during early tumor development. To investigate the role of IL-15 within the tumor microenvironment, we analyzed tumor growth in mice conditionally deleted of IL-15Rα (IL-15Rα floxed X ER-Cre Tg). Unlike IL-15Rα-/- mice, this model system allows examination of lymphocytes that have developed in the presence of IL-15 signals. In mice conditionally deleted of IL-15Rα (Tamoxifen-treated IL-15Rα floxed X ER-Cre Tg mice), tumor growth was increased compared to control mice (Tamoxifen treated IL-15Rα floxed mice). Overall, these studies suggest that IL-15 is a component of the inflammatory milieu of the tumor microenvironment that likely contributes to native anti-tumor responses. This work was supported by a seed fund from the Center for Inflammation and Cancer at the MD Anderson Cancer Center and a Cancer Prevention and Research Institute of Texas Research Training Award.

